# Structural and Functional Analysis of human lung cancer risk associated hOGG1 variant Ser326Cys in DNA repair gene by molecular dynamics simulation

**DOI:** 10.1016/j.ncrna.2019.10.002

**Published:** 2019-10-19

**Authors:** Zainularifeen Abduljaleel

**Affiliations:** a Department of Medical Genetics, Faculty of Medicine, Umm Al-Qura University, P.O. Box: 715, Makkah, 21955, Saudi Arabia; b Science and Technology Unit, Umm Al-Qura University, P.O. Box: 715, Makkah, 21955, Saudi Arabia; c Bircham International University, Av. Sierra, 2, 28691, Villanueva de La Cañada, Madrid, Spain

**Keywords:** *hOGG1*, SNP rs1052133, Variant Ser326Cys, Molecular dynamic (MD) simulation, Protein molecular modeling, Structural and functional effect, Protein structure stability

## Abstract

Oxidative damaged DNA base lesions are repaired through human 8-oxoguanine DNA glycosylase gene (hOGG1) mediated pathways. A recent report based on the meta-analysis has suggested that the DNA Repair Gene hOGG1 variant Ser326Cys [3p26.2; allele S/C in nucleotide position αHelix2 Ser⇒Cys326] was associated with Lung Cancer risk in Caucasian population will alter the level Zhong et al., 2012. To the best of our knowledge, there has not been any such comprehensive in-silico investigation that validates the functional and structural impact of non-synonymous Lung Cancer Risk Associated Protein Domain (LCRAPD) mutation Ser326Cys (rs1052133) by molecular dynamics (MD) simulation approach following prediction of hOGG1 protein before and after the mutation. Further to the native and mutant protein structures, the amino acid residue and its secondary structure were observed through a solvent accessibility model for protein stability confirmation at the point of mutation. Taken together, this study suggests that the protein functional and structural studies could be a reasonable approach for investigating the impact of nsSNPs in future studies. In addition, 4295 patients samples incorporate with the analysis that genomic data types from cBioPortal. In the result, 4295 cases (91.5%) had alterations in all genes but the frequency of alterations in our targeted hOGG1 gene was shown with and without case alteration in the ratio (Logrank Test P-Value: 0.670) Kaplan-Meier by the number of patients at risk of the survival function.

## 1 Introduction

DNA repair genes play a crucial role in maintaining the stability and integrity of the genomic DNA. More than 130 genes are involved in humans and base excision repair (BER) pathway is one of the five major DNA repair pathways [1]. The BER pathway repair lesions of the DNA bases including lesions caused by reactive oxygen species (ROS). The key enzymes of the BER pathway are DNA glycosylases. The mammalian cells have four major DNA glycosylases, including the human 8-oxoguanine DNA glycosylase (*hOGG1*), which primarily recognizes 8-oxodG but repairs other oxidized purines [2]. The *hOGG1* gene is located on the short arm of chromosome 3 (3p26.2), synthesizing the enzyme, which removes 8-oxoguanine, a derivative of a mutagenic base formed due to ROS. It breaks the glycosidic bond between the modified base and the sugar moiety, leaving an apurinic/apyrimidinic site in the DNA, which later on is incised [3]. The base excision repair (BER) pathway is the real pathway for oxidative DNA damage repair [4]. In cancer growth, the most imperative harm is brought about by responsive oxygen species is the oxidation of guanine, adenine, and thymine. The most stable is 8-hydroxyguanine (8-OH-G) [5] produced by the oxidation of guanine. 8-OH- G is highly mutagenic because it mispairs with cytosine and adenine with equal efficiency during DNA replication. This prompts an expanded recurrence of G:C to T:A trans-versions that in oncogenes [6] or tumor suppressor genes can prompt carcinogenesis. The BER pathway recognizes and repairs 8-OH-G fused into incipient DNA and it expels changed nucleosides from the pool. The principle BER segments are 8-oxoguanine DNA glycosylase *hOGG1* and hMutY homolog (Escherichia coli). The *hOGG1* involved in the immediate repair of 8-oxoguanine DNA glycosylase and MUTYH is included in the repair of A: 8-OH-G bungles because of its adenine glycosylase movement [7]. The *hOGG1* gene is located in chromosome 3p26.2 and this region of genome has been observed to be altered in various tumors, especially [8], lung [9], stomach [10], thyroid [11], laryngeal [12], colorectal [13], and pancreatic malignancy [14], indicating the loss of *hOGG1* resulting in possible tumorigenesis and loss of heterozygosity of markers [13]. The hOGG1 protein has two isoforms, α-hOGG1 (345 amino acids) and isoform β-hOGG1 (424 amino acids). The upstream 316 amino acids are common between both isoforms, however the C- terminal shows extensive alterations. The hOGG1 has two key domains; the OGG-N domain containing mitochondrial localization signal (MLS, position 9–26) that adds to the 8-oxoG-tying pocket and the HhH-GPD domain (a helix-hairpin-helix structural component followed by a Gly/Pro-rich loop and a preserved aspartic acid) containing nuclear limitation signal (NLS, 335–342) and gives both the catalytic [15] and DNA-tying functions of the DNA glycosylase. The *hOGG1* gene is highly polymorphic [16] and over 200 SNPs of this gene have previously been identified. (http://www.ncbi.nlm.nih.gov/projects/SNP). Only few of these SNPs are potentially functional and have previously been studied for their associations with cancer susceptibility. The SNP rs1052133 (Ser326Cys) in the *hOGG1* gene has been shown to alter amino acid Cys instead of Ser at codon 326 and decrease the enzyme activity. This polymorphism has previously been linked with increased lung cancer susceptibility [17]. Recently published meta-analysis report further confirmed the association of hOGG1 SNP Ser326Cys with lung cancer risk [18] and has shown increased susceptibility of lung cancer in Caucasian population. Therefore, further large-scale analysis of multiple genes expression datasets might lead to the identification of more representative gene expression signatures associated with Lung cancer predisposition. Herein, we integrated one or more independent Lung cancer genes expression datasets retrospectively, which led to the identification of a *hOGG1* genes that are associated signature associated with lung cancer systemic deterioration. We have recently [19] published hOGG1 variation (Ser326Cys; rs1052133) for breast cancer by *in-silico* studies. However, this study investigated the *in silico* studies with lung Cancer associated Ser326Cys (rs1052133) SNP variant in detail. To the best of our knowledge, there has not been any such comprehensive *in silico* investigation that validates the functional and structural impact of the hOGG1 variation (Ser326Cys; rs1052133).

This study deals with the investigation of associations between genetic mutation and phenotypic variation based on algorithms that determine the effect of an amino acid substitution that alters the protein structure and function using *in silico* methodology. Molecular dynamics (MD) simulation studies have proven valuable in increasing the understanding and gain insight to explore the effect of non-synonymous polymorphisms (nsSNPs) on the structure of a protein, particularly the influence of an amino acid change that disturbs the protein–protein interaction. Various calculations in view of arrangement and structure-based methodology have been produced to anticipate the effect of missense transformations on protein function.

The deleterious nsSNPs frequently to predict used computational procedures such as sorting intolerant (SIFT) [20], PolyPhen2 and screening for non-acceptable polymorphisms (SNAP) [21] were exploited. Based on the results of the SIFT, PolyPhen2 and SNAP analyses, we predicted a three-dimensional (3D) model structure for the hOGG1 protein domain and compared it with the mutant structure. To study the structural modifications, the native and mutant protein structures were predicted and evaluated using the structural assessment program such as ProSA-web [22]. The ProSA-web Z-score is used to examine the changes caused due to a mutation in the protein structure. The 3D confirmation computational method [23] was utilized to check the mutated site based on the range of scores between native and mutant residues. Furthermore, the computationally predicted hOGG1 domain structure carrying Ser326Cys mutation i.e. lung cancer risk associated protein domain (LCRAPD) with its native protein domain structure was compared by superimposing the two structures. Moreover, the native and mutant protein domain structures were also examined for the solvent accessibility and secondary structural arrangements.

## 2 Materials and methods

### 2.1 Dataset sources

Zhong et al., 2012 studied the polymorphism in *hOGG1* genes through meta-analysis and validated the association between the hOGG1 Ser326Cys polymorphism and lung cancer risk. They performed meta-analysis of 20 studies (8739 cases and 10385 controls) using STATA version 11.1 stratified by ethnicity, control sources, cell histotypes, and smoking status [18]. Additional 4295 patients samples incorporate with our analysis that genomic data types from the cBioPortal (http://www.cbioportal.org), which include were in somatic mutations, DNA copy-number alterations (CNAs), mRNA and microRNA (miRNA) expression, DNA methylation, protein abundance, and phosphoprotein abundance.

### 2.2 Statistical analysis

Fisher's exact test and the Mann-Whitney [24] test implied used to investigate the categorical and constant variables. We determined and compared survival curves using the Kaplan-Meier [25] method and log-rank tests [26]. Cox proportional hazards model was used to analyze associations between Clinic-Pathological symptoms and patient survival. Overall survival (OS) data was taken from the cbioportal (http://www.cbioportal.org). The representation of overall survival was the time between the procedure date when the tumor specimen was collected and the date of death or last follow-up visit [27].

### 2.3 Protein structure modeling

The SNP database for the variant rs1052133 (Ser326Cys) in the *hOGG1* gene was searched in the database of single nucleotide polymorphism (dbSNP) [28]. Detailed information about the Ser326Cys SNP was obtained from the Human Genome Variation database (HGVBASE) regarding the coding regions of the gene and the location of the mutation. The Protein Database (PDB) database revealed that the crystallographic structure of hOGG1 protein was available but only for residues up to #324, however no protein domain matched for the area where the mutation was located (Ser326Cys). The mutation (Ser326Cys) was located at position 326, therefore amino acids sequences from 296 to 345 were selected for fragment sequence alignment and for predicting a 3D protein domain structure of lung cancer risk associated protein domain (LCRAPD) based on QUARK *de novo* algorithm using *ab initio* method [29]. Consequently, the protein domain structure having the variant allele S/C (rs1052133) at codon position 326 (Ser326Cys) from the *hOGG1* gene was structurally predicted. The QUARK *ab initio* structural prediction procedure was divided into three steps; multiple feature predictions, fragment generation starting from our query sequence and decoy structure clustering along with full-atomic refinement.

### 2.4 Prediction of functional effect of tolerated and deleterious SNPs

Sorting Intolerant from Tolerant (SIFT, version 2) program identifies whether or not an amino acid substitution alters a protein function and its phenotype. SIFT program can differentiate functionally neutral and deleterious amino acid alterations and is utilized for polymorphism and mutagenesis related studies [20]. The SIFT program applies homologous sequences algorithm for detection and database based on a conserved sequence score median value 3.00. SIFT scores were classified as intolerant (0.051–0.10), borderline (0.101–0.20), and tolerant (0.201–1.00). PolyPhen2 program was used to predict the possible impact of an amino acid substitution on the structure and function of the variant protein. PolyPhen 2.0 (http://genetics.bwh.harvard.edu/pph2/index.shtml) exploits a blend of sequence and structure based features and uses naive Bayesian classifier for the identification of an amino acid substitution and the effect of mutation. In addition, the SNAP program was used to detect the effect of the variant (Ser326Cys) on the protein function [30,31]. The SNAP scores (RI >=0 binary) were translated into binary predictions effects (present/absent) and along with reliability indices (RI), where the distance from binary determination boundary (0) measures the reliability of the impact [21]. A damaging signal of a variant will indicate that the mutation is predicted to be stabilizing, otherwise it will be destabilizing. The ΔΔG values were measured in kcal/mol. Furthermore, Function Analysis and Selection Tool for Single Nucleotide Polymorphisms (FASTSNP) was also used, a web-based server, which allows users to recognize and list SNPs that are expected to have functional, effects (http://fastsnp.ibms.sinica.edu.tw).

### 2.5 Prediction of structural impact

Structural impact was performed using Have yOur Protein Defined (HOPE) program [32], which was developed by the CMBI, department of Bioinformatics of the Radboud University, USA. The program estimates the impact of a mutation on its structure. The report displays the functional contacts like metal, DNA, hydrogen bonds, ionic interactions along with the mutation impacts on the essential contact, structural areas together with motifs, domains, and trans-membrane domains. Therefore, it offers the foremost dependable method to acquire data and explores details across the "actual protein structures" based on annotated information in Universal Protein Resource (UniProt) and utilizes it by prediction by DAS-servers [33]. The information was obtained using WHAT IF web, the UniProt database and a series of DAS-servers. The structural information was obtained from the analysis of PDB ID 2XHI and annotations about this protein were accomplished from the UniProt-entry O15527 (OGG1_HUMAN).

### 2.6 Molecular dynamics simulation

CHARMM-GUI (http://www.charmm-gui.org) [34] is an online graphical user interface program to evaluate the data and molecular systems. The molecular dynamics simulations were accomplished with a 5-fs time period at a continuous temperature of 300K and along with a constant pressure of 1atm under periodic solvent boundary conditions. The Chiron program that rapidly minimizes the steric clashes in proteins using short discrete molecular dynamics (DMD) simulations was used [35], which additionally allowed the comparison of the refined structures with the predicted protein domain structure to evaluate the changes. The solvated system was not decreased nor equilibrated. The MD simulations were performed by employing the biomolecular simulation program CHARMM, widely used for macromolecular mechanic and dynamic studies, with wide-ranging assessment and management comprehensive tools for atomic coordinates along with dynamics extrapolations. Such simulations usually are based on two schemes, particularly, energy minimization and molecular dynamics, which allows an improved structure and simulated natural motion of biological macromolecules, respectively. The Gromacs exploits force field for energy minimization initially by the steepest descent; conjugate gradient and Limited-reminiscence Broyden-Fletcher-Goldfarb-Shanno (L-BFGS) approaches [36]. The free energy simulations were accomplished with a small number of explicit solvent water molecules adjacent to the solute and solvent mass was displayed as a realistic efficient solvent boundary potential (SSBP). The initial pattern of ions was confirmed using the Monte Carlo (MC) simulations through the primitive using model with van der Waals (vdW) interaction. The Particle Mesh Ewald (PME) methodology [37] was applied for electrostatics, and a 12 Å cutoff for vdW interactions. About 51M ions provided were in the simulation box by stating ions (KCl) with focusing C. The mutation (Ser326Cys) was studied using CCP4 program (QtMG) [38], and energy minimization for the 3D structures performed using Chiron server [35]. KCl was incorporated to neutralize the overall negative charge of the structures (Jo et al. 2008). A simulation of the whole protein in the water was acquired by immersing the molecular structures, in a solvent with simulation parameters, as well as energy minimization was performed to analyze of the structure. A knowledge-based server for protein structural refinement (KoBaMIN) [39] was used for the SNP variant rs1052133 (Ser326Cys) of the 3D h*Ogg1* protein domain structure to check the energy function along with the solvent environment. The solvation free energy was indicated as non-polar and electrostatic contributions; nevertheless the non-polar influence was partitioned into repulsive and dispersive contributions using exploiting the Weeks.

### 2.7 Normal mode (NM)-Based geometric simulation of hOGG1 domain

The protein conformational changes and normal modes were computed for the LCRAPD (hOGG1) structure through the stringent cluster normal-mode analysis (RCNMA) module [40]. The NMSim module primarily obtained the structure based on low-frequency normal modes and then generated a reliable chemically valid conformation from the predicted structure [41]. The RCNMA and NMSim programs were incorporated with supercomputing cluster with capability of high nodes clustering. The LCRAPD structure was used for the NM simulation for before and after the alteration for the biologically relevant conformational transitions in the hOGG1 protein domain established on the following fixed parameters: the radius of gyration motions, E-cutoff for h-bonds (kcal/mol): −1, Hydrophobic cutoff (Å) and method: 0.35, NMSim No. Of trajectories: 1, No. Of cycles: 1, Side-chain distortions: 0.3, No. Of simulation cycle: 500, output frequency: 1, Nm mode range: 1–50, ROG mode: 1, step size: 0.5. RCNMA cutoff for c-alpha atom (Å): 10. The estimated runtime was 66.84 h (Performing simulation cycle no of 1-500atoms/1–5). The RMSD and RMSF values were calculated subsequently superimposing the modeled conformations onto the native LCRAPD structure.

## 3 Results

### 3.1 Genomic alterations associated with hOGG1 - genes in lung adenocarcinoma

**Fig. 1:**
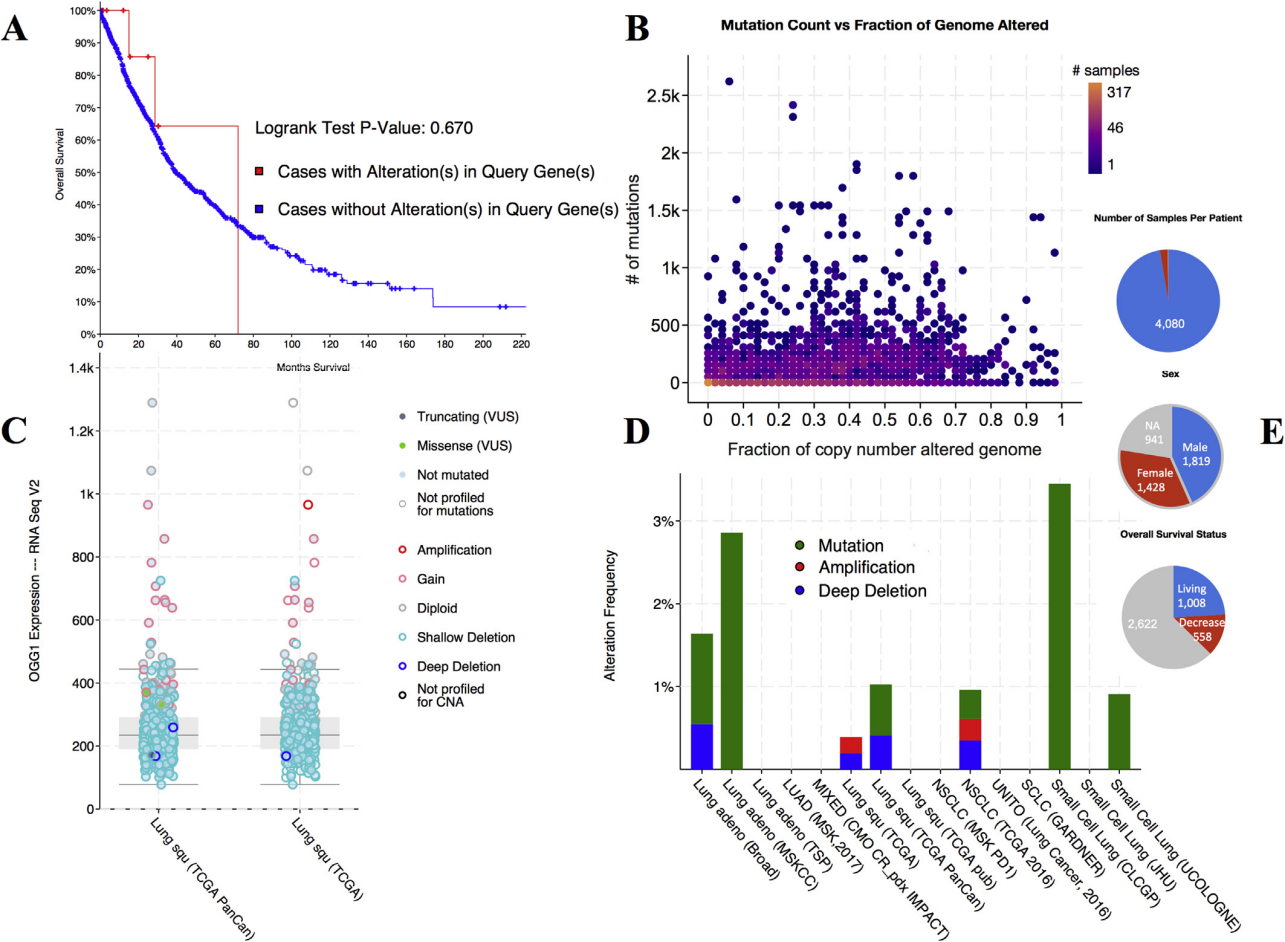
A-E. (A) The overall number of samples survival of case with and without alteration (Logrank Test P-Value: 0.670). (B) Overview of clinical attributes and a scatter plot of mutation count versus fraction of genome altered for each case in the TCGA endometrial cancer study. (C) ERBB2 mRNA expression is increased in samples with DNA amplification, and ERBB2 protein abundance was higher in samples with increased mRNA. A plot showed the relationship between ERBB2 mRNA abundance and CNA in the ERBB2 gene of Ogg1 in tumors from the selected lung cancer. (D) Different types of lung cancer with alteration shown in a different frequency (E) The overall survival for the total number of average infected both male and females, A status of the patients condition living and decrease and the number of samples per patient.

The mRNA versus copy-number shown a box-and-whisker plot to mRNA expression of a gene plotted in relation to its copy-number alteration in each Lung adenocarcinoma dataset sample total from 4295. Copy-number preserve be either were in homozygously deleted, heterozygously deleted, diploid, gained amplification with relatively number of copies, or amplified. The mRNA-versus-DNA methylation of a scatter plot of mRNA expression compared with DNA methylation of genes across from all selected samples. A methylation beta-value is an estimate for the methylation level of a CpG locus by using the ratio of intensities between methylated and unmethylated alleles. The hOGG1 protein level versus mRNA scatter plot of protein abundance compared with mRNA abundance for a gene across all selected samples. In this study, 4295 cases (91.5%) had alterations in all genes; the frequency of alterations in our targeted *hOGG1* gene was shown with and without case alteration in the ratio (Logrank Test P-Value: 0.670) by Kaplan-Meier estimate in Fig. 1A–E. For most alterations were classified as deep deletions, with a few cases of truncating mutations. For MutyH (51%), the majority of alterations were amplifications, with a small fraction of missense mutations. For PTEN (27%) and BRCA1 (22%), the gene changes included deep deletions and truncating, missense and inframe mutations.

### 3.2 MD simulation and solvent accessibility

**Fig. 2:**
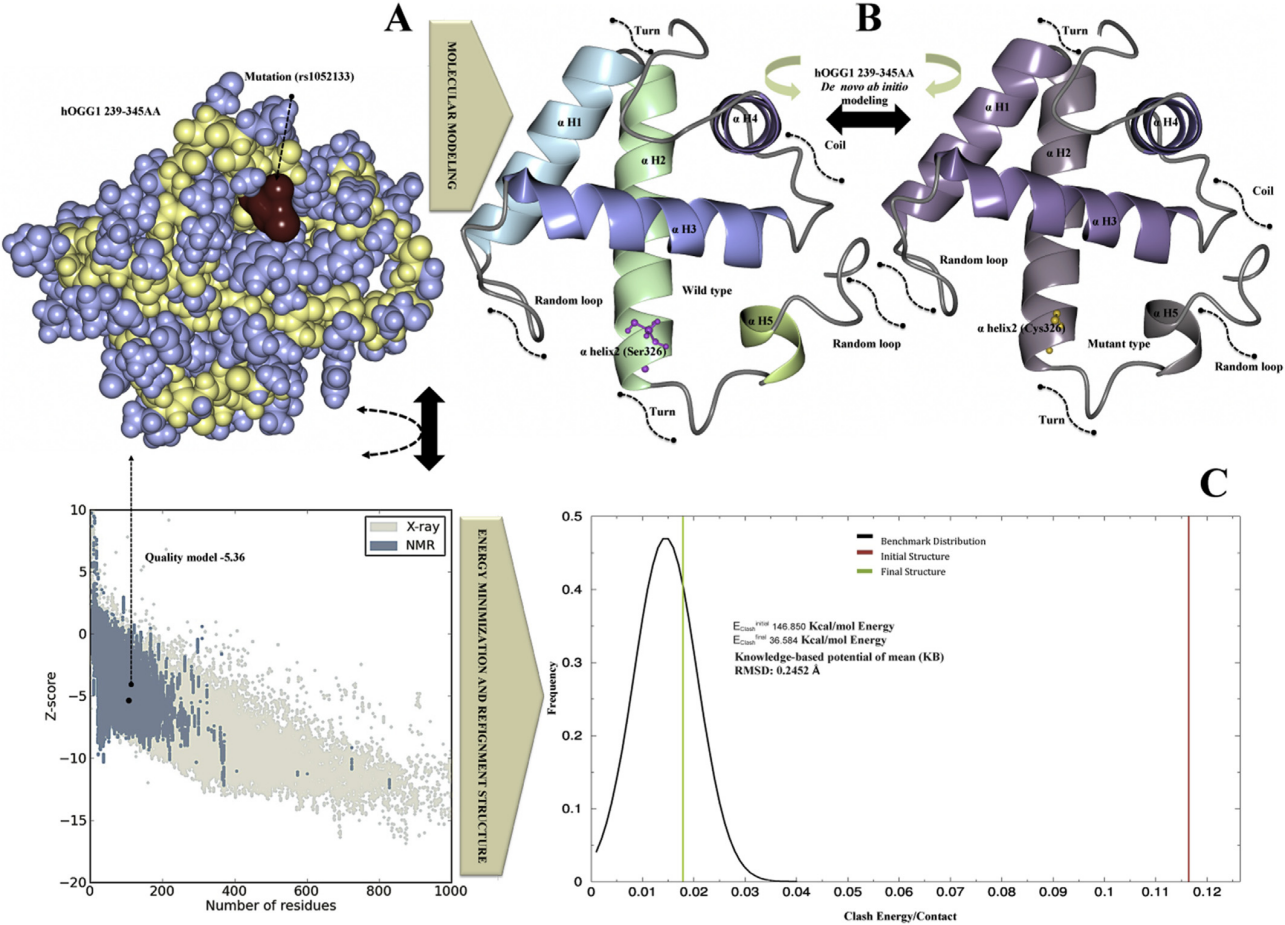
A-C. **The LCRPD structure modeling validation, energy minimization and refinement** **A)** The predicted protein structural model was based on sphere model for hOGG1 239–345 amino acid residues (blue and yellow color) the location of mutated residue is shown in red. The Z-score, which indicates overall model quality was −5.36 for LCRPD (black color). The Z-score plot from different sources (X-ray, NMR) was distinguished by various colors (X-ray in sandal; NMR in blue). **B**) The structure of wild type LCRPD structure with αhelix2 Cys326 shown in blue and green colors, whereas the mutant structure having αhelix2 Ser326 is shown in violet and grey colors. **C)** The LCRPD structural refinement and energy minimization were performed using MD simulation where the benchmark distributions is shown in black color, initial structure (146.850 kcal/mol Energy) is shown in red, whereas the final structure (36.584 kcal/mol Energy) is shown in green color. The RMSD was 0.2452 Å.

**Fig. 3:**
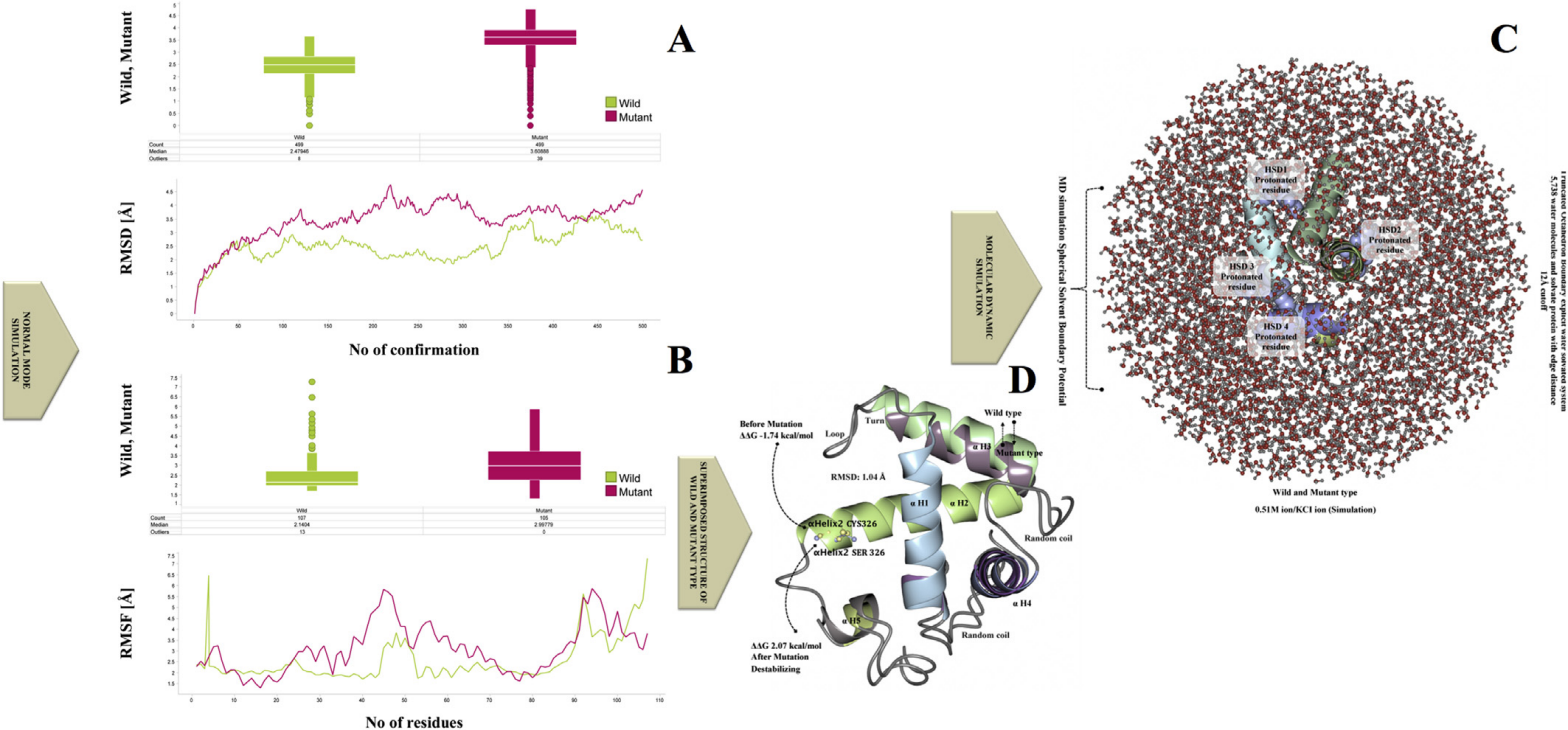
A-D. **Protein conformational changes via simulation** **A)** The graph is showing the Cα atom RMSD of the initial structure over the trajectory obtained for a ROG-guided NMSim for LCRPD structure before and after mutation. The wild type is shown in green and the mutant type is shown in red color. **B)** The graph is showing the Cα atom RMSF over the trajectory obtained for a ROG-guided NMSim for the modeled protein domain before (green) and after (red) mutation. **C)** The LCRPD structure was shown via MD simulation with truncated octahedron boundary explicit water solvated molecule and hydrogen atoms. The side chain of a histidine residue was interacting with the hydrogen bond along with surrounding molecules and the δ nitrogen of the histidine (HSD1-4) was a protonated residue. **D**) The LCRPD structure before the mutation was αhelix2 Ser→Cys326 (S→C) (blue and green color) (ΔΔG -1.74 kcal/mol), whereas after the destabilizing mutation it was changed αhelix2Cys→Ser326 (C→S) (ΔΔG 2.07 kcal/mol) (violet and grey color). The two structures (wild and mutant) were superimposed showing RMSD 1.04.

Point mutation causing amino acid modification may significantly alter the stability of the protein structure; therefore, protein structural data provide useful information for a comprehensive understanding of its functionality. The information about the SNP variant Ser326Cys) in the *hOGG1* gene was obtained from the database of single nucleotide polymorphism (dbSNP) (http://www.ncbi.nlm.nih.gov/SNP) [28]. Further information about the coding regions of the gene and the location of the Ser326Cys SNP variant was collected from Human Genome Variation database (HGVBASE) (www.hgvs.org/). The PDB database search revealed no protein domain matching the hOGG1 protein structure in the area of the Ser326Cys mutation. Crystallographic structure of hOGG1 protein was available for residues up to 324, but not for the area where the variant Ser326Cys was located i.e. position 326. Hence, sequences from 296 to 345 amino acids in hOGG1 were selected for fragment sequence alignment for the structure prediction based on QUARK *de novo* algorithm using *ab initio* method. The mutant amino acid position was αHelix2-Ser326Cys where Cystine was the mutant residue instead of Serine. The QUARK *de novo* algorithm resulted in ten best models based on the C-score for each model. The selected model consisted of the largest cluster and showed the best C-score among the top ten templates generated (Fig. 2A). The protein structure confirmation and quality of the native protein structure were obtained by ProSA program (https://prosa.services.came.sbg.ac.at/prosa.php). The overall quality of the model indicated a Z-score of −5.36. The predicted LCRAPD before and after the mutation (αHelix2 Ser⇒Cys326) was obtained using SWISS-PORT and CCP4 (QtMG), showed the best estimated TM-score, 1: 0.4121 ± 0.0833 based on I-Tasser results (Fig. 2A and 2B). The native and mutant structures of the LCRAPD were examined based on the RMSD using NOMAD-Ref Gromacs and KoBaMIN. The results showed that LCRAPD after Ser326Cys mutation resulted in higher energy value compared to the native structure without mutation (Fig. 2C). The KoBaMIN program was used to minimize the energy of the mutated LCRAPD structure (−146.850 kJ/mol; score 0.80) compared to the native structure energy (36.584 kJ/mol; score: −3.33). After energy minimization refinements the structure based on the potential of mean force resulted in 0.2452 Å RMSD. The normal mode simulation (NMSim) program was used for observing the multi-scale modeling of protein conformational changes in native and mutant protein structures. The RMSD graph was constructed using Cα atom of LCRAPD structure showing a number of conformations between the native and the mutant structures. The two graphs (Fig. 3A and 3B) demonstrate the conformational changes of the mutant and native LCRAPD structures. The graph 3A indicates the Ca α atom effect on the RMSD of native and the mutant structures over the trajectory, whereas the graph 3B exhibits the Ca α atom of these two structures based on RMSF over the trajectory. The RMSD and RMSF were calculated by superimposing the modeled conformations onto the wild LCRAPD structure with respect to the Ca α atoms. The CHARMM program was used for the MD simulations to observe the consequences of the mutation by comparing the native and mutant structures under solvation. The results indicate that the solvate accumulated successfully around the LCRAPD structure with water molecules and showed fully solvated structure with an edge distance of 10.0 (Fig. 3B). The results showed that the LCRAPD before mutation showed a ΔΔG -1.74 kcal/mol energy which was stabilizing, however after the Cys326 mutation, the energy was higher i.e., ΔΔG 2.07 kcal/mol and was destabilizing (Fig. 3C). The native and mutant LCRAPD structures were also superimposed and exhibited an RMSD of 1.04 Å.

### 3.3 Structural implications of the disease-causing variant

**Fig. 4:**
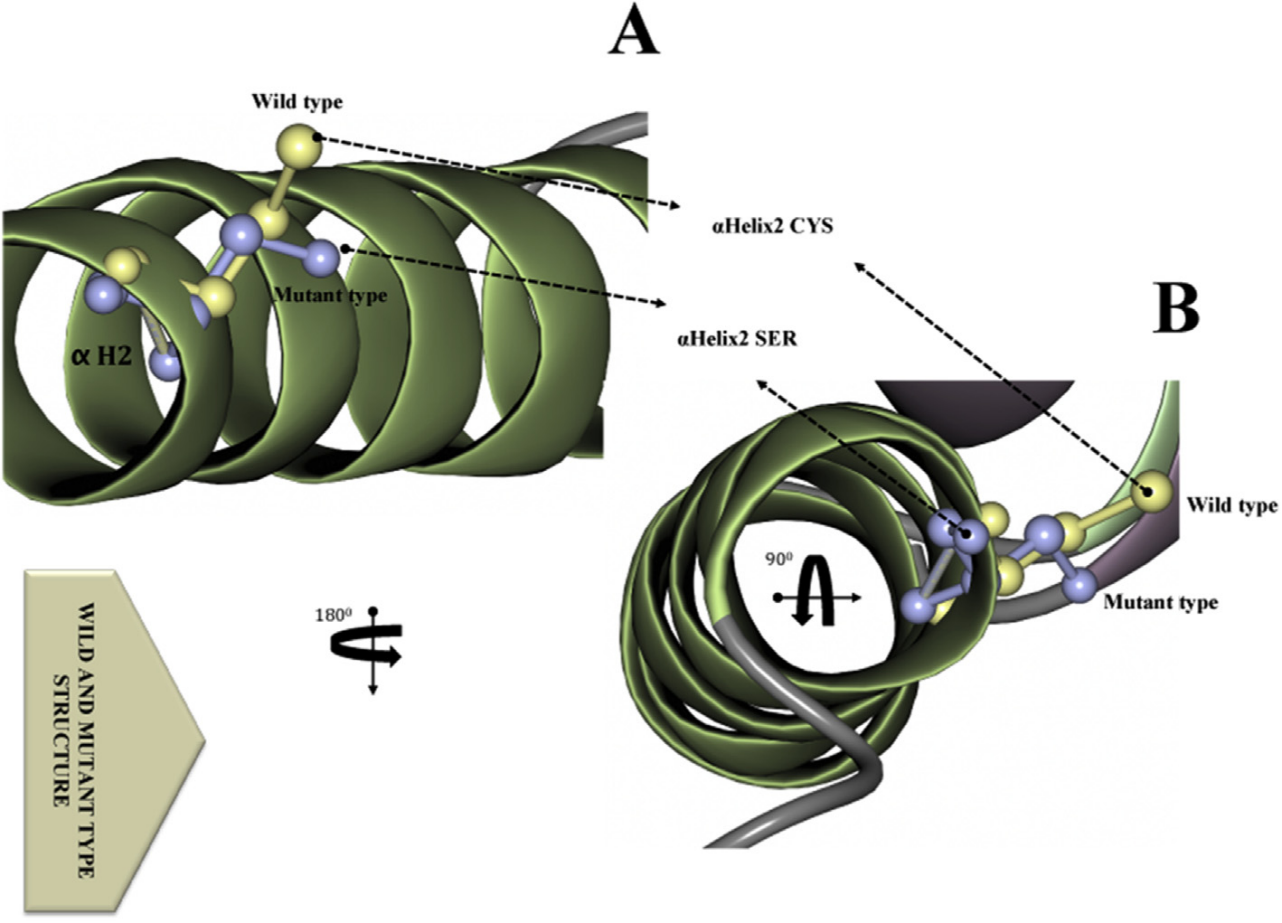
A-B. **The superimposed native and mutant LCRPD structures with different orientations** **A-B)** The mutant (green) and wild type (violet) LCRPD structures with particular residue shown in yellow (wild type, αhelix2 CYS,) and grey (mutant type, αhelix2 SER).

The native and the mutant residues differed in physical and functional properties (Fig. 4A–B). The results showed that the Ser326Cys mutation varied from the wild residue in specific size, charge, and hydrophobicity values. Residues in the vicinity of the mutated residue of Cys when annotated with the UniProt database revealed to be a binding site, hence, the mutation may perhaps effect the LCRAPD structure at the site of mutation and will consequently affect the binding site. The wild residue in the 3D structure was situated in its preferred secondary structure, which was a turn. However, the mutant residue altered the secondary structure; and the local conformation became destabilized and expected to be damaging to the protein. The Ser326Cys mutation was located in an area known to be splicing variants isoforms "2B and 2C". The mutant Cys residue accompanied along with other types of residues formerly have been perceived at this location in other homologous sequences. The mutant residue was buried in the core LCRAPD domain and caused a disturbance in the structure. The wild type and mutant residues also differed in their hydrophobicity and resulted in the loss of hydrogen bonds of the core region of the domain, hence will eventually disturb the correct protein folding conformation. Support Vector Machine (SVM) was applied for numerous classification tasks. The SNPs 3D database provides SVM profile score for the functional effect of deleterious or non-deleterious nsSNPs. The observed SVM score for the Ser326Cys variant was −0.82 and showed its molecular effect on the protein structure under salt bridge (Table 1).

**Table 1:** Structural effects of non-synonymous SNPs.

Molecular Functional Effects of non-synonymous SNPs						
Refseq accession	SNP	SNP ID	SVM	SVM structure	Molecular effect	Frequency
NP 002533	P27T	rs11548133	−0.04	0.46	On the protein surface	–
	A25S	rs19050550	1.22	0.94	On the protein surface	–
	R229Q	rs1805373	−0.61	−0.46	Hydrogen Bond Lost and Salt Bridge Lost	0.03
	A288V	rs3219012	0.59	1.16	On the protein surface	0.00
	S320T	rs1801128	1.21	−1.15	Salt Bridge Lost	0.01
	D322N	rs3219014	−0.07	−1.06	–	–
	S326C	rs1052133	−0.82		Salt Bridge Lost	0.29
NP 058212	P27T	rs11548133	−0.01	0.46	On the protein surface	–
	A85S	rs17050550	1.13	0.94	On the protein surface	0.03
	A288V	rs3219012	0.70	1.16	On the protein surface	0.00
NP 058213	P27T	rs11548133	−0.05	0.54	On the protein surface	–
	A85S	rs17050550	1.24	0.98	On the protein surface	–
	R229Q	rs1805373	−0.68	−0.04	Hydrogen Bond Lost and Salt Bridge Lost	0.03
	A288V	rs3219012	0.76	1.14	On the protein surface	0.00
	Y321H	rs3219013	−0.10		–	0.00
NP 058214	P27T	rs11548133	−0.06	0.46	On the protein surface	–
	R229Q	rs1805373	−1.02	−0.46	Hydrogen Bond Lost and Salt Bridge Lost	0.03
	A288V	rs3219012	1.12	1.16	On the protein surface	0.00
NP 058434	P27T	rs11548133	−0.35	0.46	On the protein surface	–
	A85S	rs17050550	1.19	0.94	On the protein surface	–
	R229Q	rs1805373	−0.89	−0.25	Hydrogen Bond Lost	0.03
NP 058436	P27T	rs11548133	0.23	0.46	On the protein surface	–
	A85S	rs17050550	0.99	0.94	On the protein surface	–
NP 058437	P27T	rs11548133	−0.04	0.56	On the protein surface	–
	A85S	rs17050550	−0.04	0.46	On the protein surface	–
	R229Q	rs1805373	−0.80	−0.46	Hydrogen Bond Lost and Salt Bridge Lost	0.03
	A22V	rs3219012	1.00	1.16	On the protein surface	0.00
NP 058438	P27T	rs11548133	1.11	0.94	On the protein surface	–
	R229Q	rs1805373	−0.99	−0.46	Salt Bridge Lost	0.03
	A288V	rs3219012	0.91	1.16	On the protein surface	0.00

### 3.4 Functional implications of the disease-causing variant

**Fig. 5:**
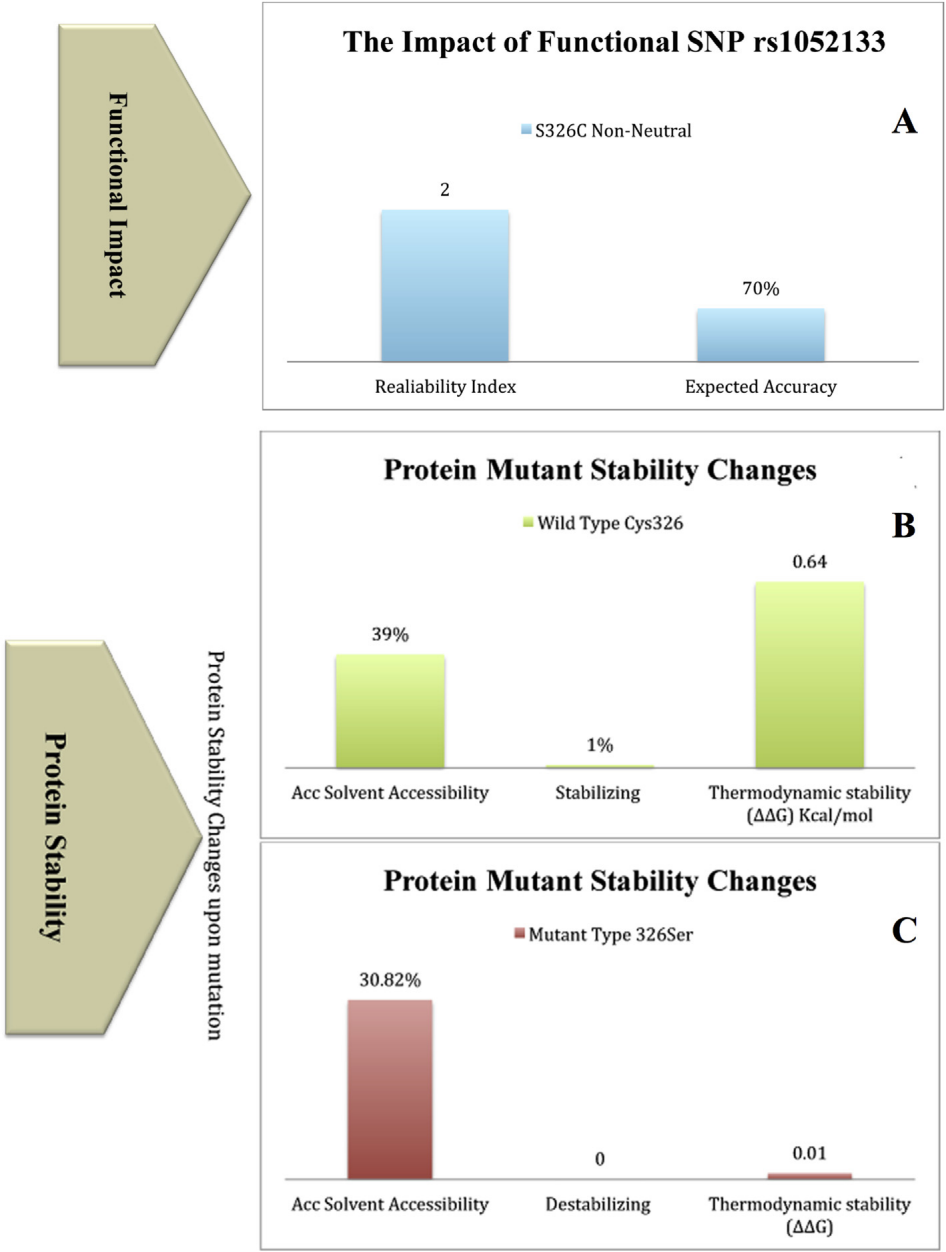
*A-C* **A)** Effects of single amino acid substitutions on protein function. **B–C)** The protein stability changes upon mutation.

The SNPs have been known to represent the largest number of all genetic variations. We utilized SNAP method to identify its influence on the protein functionality. The result if shown as "neutral” will not be functionally different from the wild type, however if it is "non-neutral" then the mutant based on the output score will have an impact on the phenotype and perhaps be deleterious (Fig. 5A). The SNAP scores (RI >=0 binary) translated into binary predictions effects (present/absent) along with the reliability indices (RI) showed an expected 70% risk for the *hOGG1*. In addition, SIFT program was used to examine the tolerance and intolerance of a substitution form all other SNPs of the *hOGG1* gene from 301 to 345 amino acid residues of LCRAPD and showed an intolerance index score 0.05 (Table 2). The intolerance threshold Seq-Rep indicated that the sequence has one of the basic amino acids, where as a small fraction shows that the site was severely gapped or unalienable. The SIFT scores were classified as tolerant (0.201–1.00) or intolerant (0.051–0.10) and borderline (0.101–0.20). About 45 out of 500 nsSNPs, showed extremely deleterious tolerance index score 0.00, 3 showed 0.02 score and other 3 had a 0.04 score, whereas the other 39 had 0.01 score signifying that the SNP in the *hOGG1* gene will have an impact on the protein function. Furthermore, the PolyPhen program also predicted that the amino acid substitution would probably have an impact on the structure and function of the protein when 12 nsSNPs protein sequences were analyzed. The results were based on the position-specific independent score (PSIC) differences (PSIC SD; ≥ 1.5 w) interpreting that an amino acid substitution is considered to be damaging and most likely will have an impact. Our results showed that out of the 12 nsSNPs, five (rs1805373, rs55667729, rs9824261, rs1052133 and rs1052134) were damaging with PSIC SD score ≥ 2.5, twenty with PSIC SD score ≥ 2.0 and one with PSIC SD score ≥ 0.01 and were also shown to be deleterious following SIFT analysis. Based on the PSIC SD as well as SIFT scores, one of the nsSNPs rs1052133 that showed SIFT damaging score 0.05 and PSIC score 1.2, was selected for further analysis. Furthermore, to identify the nsSNP showing a functional effect, FASTSNP was also applied for exploring the nsSNPs influence on the protein's cellular and molecular function such as transcription and splice regulation. The prioritizing method was used for SNPs in transcripts filtering. There were 21 transcript SNPs however, only one transcript (ID: ENST00000344629; Refseq mRNA) was selected. The result of first filtration showed 30 SNPs in 5′-Upstream, 8 SNPs in 5′-UTR, 74 SNPs in intronic 4 SNPs in 3′-UTR, 38 SNPs in 3′-downstream and 12 SNPs in the coding region. The functional significance of *hOGG1* nsSNPs is also shown in the Table 3.

**Table 2:**
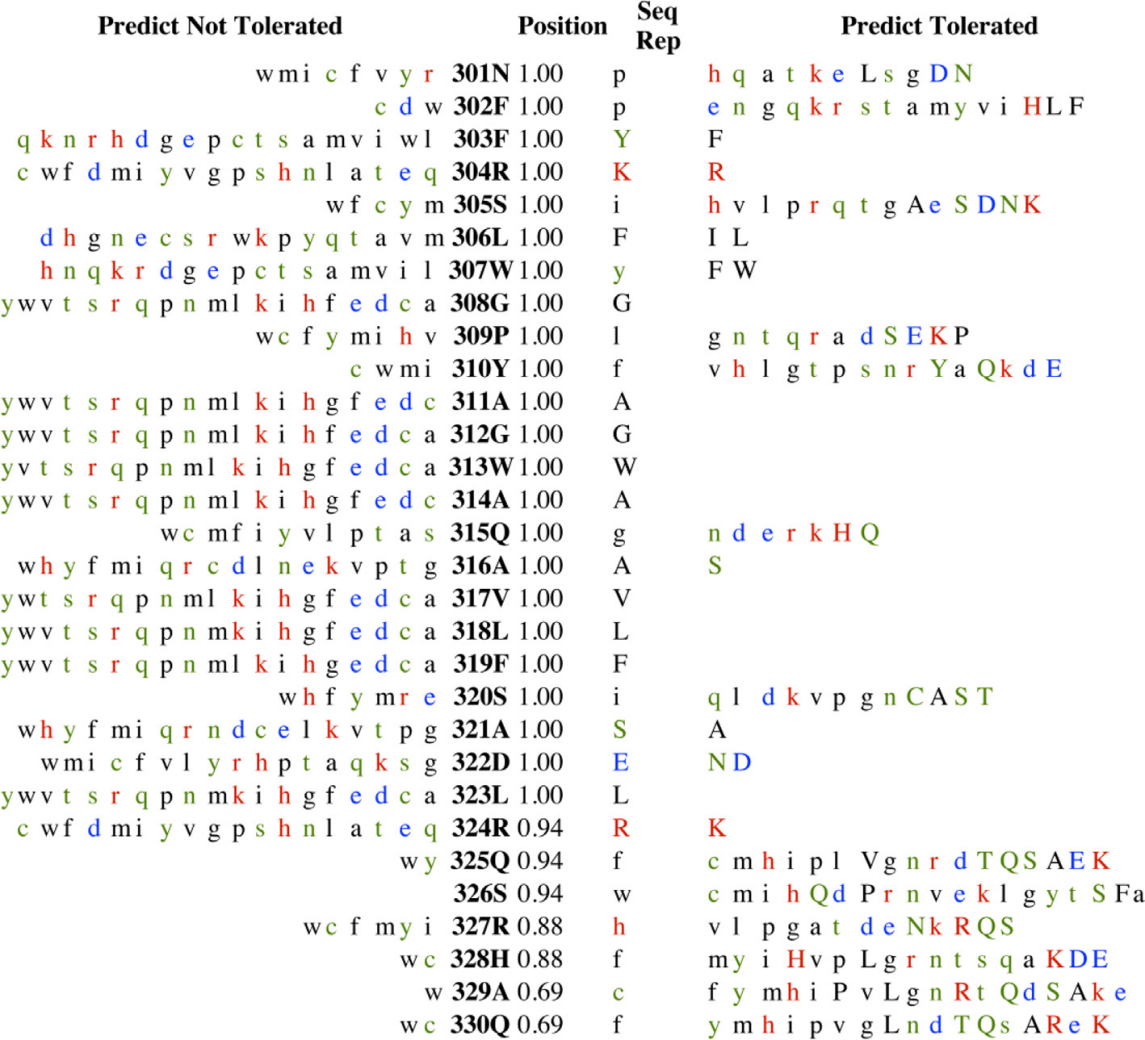
Protein Functional Impact.

**Table 3:** A functional SNPs analysis based on filtering.

A Functional SNPs Analysis													
SNP ID (rs)	Functional Effects	Risk	Gene	RefSeq mRNA	Allele	Region	Transcript start site	AA. Pos	Chr.Pos	Polyphen2	PSIC Score	SIFT	SubPSEC Score
rs1805373	Missense (non-conservative); Splicing regulation	Medium-High (3–4)	OGG1	ENST00000344629	G/A	Coding	4880	229	Chr3: 9796508	Possibly damaging	2.091	TOLERATED	1.00
rs11548133	Missense (conservative); Splicing regulation	Low-Medium (2–3)	OGG1	ENST00000344629	C/A	Coding	421	27	Chr3: 9798773	benign	0.962	DAMAGED	0.26
rs17050550	Missense (conservative)	Low-Medium (2–3)	OGG1	ENST00000344629	G/T	Coding	1116	85	Chr3: 9792744	benign	0.193	DAMAGED	0.01
rs1801127	Sense/synonymous; Splicing regulation	Low-Medium (2–3)	OGG1	ENST00000344629	G/A	Coding	1157	98	Chr3: 9792785	benign	–	TOLERATED	–
rs56053615	Missense (conservative); Splicing regulation	Low-Medium (2–3)	OGG1	ENST00000344629	G/A	Coding	1901	154	Chr3: 9793529	benign	–	TOLERATED	0.00
rs55667729	Missense (conservative); Splicing regulation	Low-Medium (2–3)	OGG1	ENST00000344629	G/C	Coding	4882	230	Chr3: 9796510	Possibly damaging	–	DAMAGED	0.04
rs3219012	Missense (conservative); Splicing regulation	Low-Medium (2–3)	OGG1	ENST00000344629	C/T	Coding	6642	288	Chr3: 9798270	benign	1.071	DAMAGED	0.00
rs1801128	Missense (conservative)	Low-Medium (2–3)	OGG1	ENST00000344629	G/C	Coding	7127	320	Chr3: 9798755	benign	0.675	DAMAGED	0.00
rs3219014	Missense (conservative); Splicing regulation	Low-Medium (2–3)	OGG1	ENST00000344629	G/A	Coding	7132	322	Chr3: 9798760	benign	0.200	TOLERATED	0.08
rs9824261	Sense/synonymous; Splicing regulation	Low-Medium (2–3)	OGG1	ENST00000344629	C/T	Coding	7135	323	Chr3: 9798763	Possibly damaging	–	TOLERATED	–
rs1052133	Missense (non-conservative); Splicing regulation	Low-Medium (2–3)	OGG1	ENST00000344629	C/G	Coding	7145	326	Chr3: 9798773	Possibly damaging	1.212	DAMAGED	0.05
rs1052134	Missense (conservative); Splicing regulation	Low-Medium (2–3)	OGG1	ENST00000344629	C/G	Coding	7146	326	Chr3: 9798774	Possibly damaging	1.691	TOLERATED	0.08

### 3.5 Impact of Ser326Cys mutation on the protein stability

To observe the thermodynamic protein stability changes as a result of the Ser326Cys mutation in the protein, PopMuSic-2.0 was utilized based on the statistical potentials of linear sequence coefficients to determine the solvent accessibility model following the alternation. The results showed that altering amino acid Ser to Cys at position 326 will cause excessive folding free energy (ΔΔG = 0.64 kcal/mol) in the mutant structure compared to the native LCRAPD structure (ΔΔG = 0.01 kcal/mol). The mutation caused a significant (r^2^ = 0.8) disturbance in the protein folding particularly in the region of the mutation and resulted in noticeable stability changes. The solvent accessibility (Acc) modeling altered LCRAPD structure, indicating that the Acc for the mutant (Ser326Cys) was 39.01 (31%) whereas for the native structure it was 30.82 (30%) (Fig. 5B–C). The structural weakness was considerably higher than the normal, indicating that a mutant site may alter the proper function of a protein when it becomes un-stabilized.

## 4 Discussion

Several severe ailments including cancer are due to oxidative stress. When the oxidative stress surpasses beyond the usual protective mechanism's capability, the reactive oxygen species causes modifications in the DNA such as d 8-oxo-guanine base alterations that may lead to carcinogenesis. The carcinogenesis in the lung tissue may be due to tissue injury where the ROS can react with the DNA and generate genomic mutations [42]. One of the key mechanisms that preserve the genomic stability is the base excision repair (BER) and any alterations in this function will result in 8-OHG lesion accumulations including other DNA base lesions, which will have an impact on the initiation and progression of cancer [43]. The hOGG1 is an important enzyme of the BER pathway because of its role in the initial excision of the regularly occurring oxidative damage of the DNA 8-OHG base [44]. The hOGG1 is an 8-oxoguanine DNA glycosylase/AP lyase responsible for the elimination of 8-OHG from DNA [45]. Among the several polymorphisms that have been observed in the *hOGG1* gene, single nucleotide polymorphism at codon 326 (Ser326Cys) is the most studied one. Homozygous carriers of the Ser326Cys variants of the *hOGG1* gene have shown to lessen the repair capacity for the oxidized DNA lesions [46]. It has been suggested that the Ser326Cys (rs1052133) polymorphism of *hOGG1is* due to the oxidative damaged of DNA and its repair activity through dominant and additive effects [47]. We have recently reported the involvement of Ser326Cys genotype and increased risk of breast cancer in the Saudi individuals and also studied the structural consequences of hOGG1 variant Ser326Cys through structural prediction and *in silico* computational analysis [19]. The results further showed that the variant Ser326Cys probably disrupt the protein structure and may result in the malfunction of the hOGG1 protein.

Newly, remodeling controls the expressions of many genes are associated as tumor suppressors in Lung adenocarcinoma [48] and in other cancers [49]. Importantly, variations in copy number and somatic mutations in Ogg1 are present in many types of cancer [30] (http://www.cbioportal.org/public-portal/). Additional considerations are required to elucidate how hOGG1 contributes to Lung adenocarcinoma susceptibility. For example, the risk (72Pro) allele of the TP53-Arg72Pro SNP encodes a protein with weaker apoptotic activity of the 72Arg allele that enables increased survival of DNA-damaged cells while the risk (326Cys) allele of the Ser326Cys SNP in hOGG1 encodes a DNA glycosylase with weaker activity in the repair of oxidative promutagenic base damage, 8-hydroxygua- nine, produced by tobacco and other carcinogens than that of the 326Ser allele [50].

In this study for the first time the functional and structural impact of the Ser326Cys variant through *in-silico* approaches. Based on a recently published meta-analysis study, the variant Ser326Cys [3p26.2; allele S/C in nucleotide position αHelix2 Ser⇒Cys326] of *hOGG1* gene has shown that the mutated variant is associated with the increased lung cancer risk in the Caucasian population [18].

It has been observed that some point mutations may cause a change in the amino acid, which will alter the stability of a protein structure. Hence, the mutant protein's structural information could provide useful information to understand its functionality. The SNP data for the Ser326Cys mutation was obtained from databases dbSNP and HGVBASE, which revealed that the mutation was present in the exonic region of *hOGG1* gene causing a missense modification. Upon the PDB database search, the hOGG1 protein showed a structural domain up to only 324 residues. Because our the mutation (Ser326Cys) was located at residue 326 and there was no hOGG1 protein structural domain available for that region in database, therefore a protein structural domain spanning the mutant residue (Ser326Cys) at codon position, a 345 amino acid long stretch of peptide sequence starting from 296 to 345 amino acids (LCRAPD) was predicted using QUARK *de novo* algorithm [29] followed by the prediction of altered predicted structure using SWISS-PORT and CCP4 programs. In addition, the NOMAD-Ref Gromacs and KoBaMIN programs were used for the native and mutant protein structures based on the RMSD minimizations energy values. Once the native protein structure through energy minimization was performed by KoBaMIN program a pertinent protein domain structure was generated. The mutation impact on predicted protein structure was then also observed through the molecular dynamic simulations for native and mutated structures under appropriate solvent conditions.

The results confirmed the previous claim that the Cys326 residues may likely be involved in protein function and are the possible candidates for OH-induced modifications and Gαi2 activation [51]. The functional and structural studies evaluated the effect of the mutated “Cys” residue and showed that as a result of mutation the size, charge, and hydrophobicity values of the protein structure were altered. Residues in the area of the mutated residue (Cys) when annotated indicated to be a binding site; thus as a consequence Ser326Cys mutation could affect the local structure and the binding site. The local conformation was destabilizing because the mutation was located in a region with known splice variants such as "In isoform 2C” and "In isoform 2B”. It is therefore, expected that the mutation will likely be damaging to the protein due to the loss of hydrogen bonds and as a result of alterations in its folding pattern.

The SIFT program in this study was used to observe the effect of amino acid substitution on protein function. The SIFT predictions were made on the basis of amino acid conservation among sequence alignments of the closely related sequences. The SIFT extrapolations for the sites from 301 to 345 residues of the LCRAPD structure were studied and a small fraction showed that the location was severely gapped or unalienable, therefore a prediction low quality extrapolation was observed at this position, when results were obtained based on previously acknowledged classification [52]. The SIFT scores for the 500 nsSNPs indicated that 45 nsSNPs were highly deleterious with tolerance index score 0.00A. The variation within the functional domain in both oxygen binding and protein interactive region indicated that it will likely have an impact on the protein function and structure stability. The results endorsed that the accurate prediction of a protein stability alterations due to single amino acid mutations proved to be important to understand the protein's structural and functional effects. Polymorphism in DNA repair genes may alter protein function and an individual's ability to repair the damaged DNA; therefore, any error in the DNA repair efficiency will lead to cause a critical genetic mutagenesis and consequently will result in carcinogenesis [53]. Our results, based on the coefficients determined by solvent accessibility of the mutated residue (Ser326Cys), showed that the thermodynamic properties of the protein stability changes will have an impact on the protein structure and function. The results yielded excessive-folding free energy (ΔΔG = 0.01 kcal/mol) due to Ser326Cys amino acid alteration compared to the normal folding (Cys326Ser) with free energy (ΔΔG = 0.64 kcal/mol), causing it to be destabilizing. The dataset of known catalytic sites will be considerably bigger than the normal and will result in structural changes, hence will affect the function and the stability of the protein due to Ser326Cys amino acid alteration. Overall these studies, computationally predicted hOGG1 structure and compared the mutant protein carrying Ser326Cys mutation with the native protein structure by superimposed. In addition, we also examined the native and mutant protein structure for solvent accessibility and secondary structures. As well as limitation parameter of these studies in future to continue relating to elucidating the role of this SNP in treatment response would be helpful for the better management of this disease.

## 5 Conclusion

The functional and structural impact of disease causing hOGG1 SNPs variant Ser326Cys was studied using computational as well as bioinformatics strategies and the predicted normal and mutated protein structures were compared. Our results proved and confirmed the previously reported finding related to the Ser326Cys variant through detailed computational and bioinformatics approaches. This study implies that the current structural and functional prediction procedures used in this analysis are valuable tools for selecting a set of probable disease-associated SNPs and to observe their relevance to a particular disease. Nonetheless, supplementary long-term follow-up investigations may possibly be a prerequisite to estimate the survival rates that are associated with the risk allele.

## Declaration of competing interest

The authors have no conflicts of interest to declare.
